# Andrographolide relieved pathological pain generated by spared nerve injury model in mice

**DOI:** 10.1080/13880209.2018.1426614

**Published:** 2018-02-01

**Authors:** Huang-Chi Wang, Hsin-Sheng Tsay, Hui-Nung Shih, Yi-An Chen, Kai-Ming Chang, Dinesh Chandra Agrawal, Siendong Huang, Yi-Lo Lin, Meng-Jen Lee

**Affiliations:** a Department of Applied Chemistry, Chaoyang University of Technology, Taichung, Taiwan, ROC;; b Department of Research, Koo Foundation Sun Yat-Sen Cancer Center, Taipei, Taiwan, ROC;; c Department of Applied Mathematics, National Dong Hwa University, Hualien, Taiwan, ROC;; d Graduate Institute of Veterinary Pathobiology, National Chung Hsing University, Taichung, Taiwan, ROC

**Keywords:** Allodynia, *Andrographis paniculata*, astrocytes, IL-1, von Frey test

## Abstract

**Context:** Andrographolide (Andro), found in large quantities in *Andrographis paniculata* Nees (Acanthaceae), is anti-inflammatory, especially in the central nervous system (CNS) glia.

**Objective:** The objective of this study is to test Andro’s ability to reduce allodynia in a spared nerve injury model.

**Material and methods:** Male 30 g BalbC mice were divided into four groups: (1) Sham-operated control (Sham-group); (2) nerve injured and treated with saline (Saline-group); (3) nerve injured and treated with Andro (Andro-group); (4) nerve injured and treated with non-steroidal anti-inflammatory drugs (NSAIDS) (NSAIDS-group). Andro or NSAIDS (diclofenac salt) were injected intraperitoneally at 5 mg/kg body weight daily. Mechanical allodynia was assessed by von Frey tests at 3, 7, and 14 d. For immunohistochemical analysis, samples were collected at 7 d.

**Results:** The threshold for inducing allodynia increased and the response percentage reduced in the Andro-group when compared with the Saline-group, as well as when compared with NSAIDS groups throughout 3–14 d. The ratio of threshold for OP-Andro/OP-saline and for OP-Andro/OP-NSAIDS groups was 20.42 and 11.67 at 14 d, respectively. The ratio of response percentage for OP-Andro/OP-saline and for OP-Andro/OP-NSAIDS was 0.32 and 0.39 at 14 d, respectively. Interleukin-1 (IL-1) immunostaining in the spinal cord was reduced in the Andro-group. Astrocytic activities were not significantly reduced in the Andro-group compared with the Saline-group at 7 d post-operation (PO)

**Conclusions:** Andro reduced mechanical allodynia more than NSAIDS at the same concentration, and the observed behaviour was associated with a reduction in inflammatory cytokine produced in the spinal cord.

## Introduction

Andrographolide (Andro) is found in large quantity in *Andrographis paniculata* Nees (Acanthaceae) (Koteswara et al. 2004). *Andrographis paniculata* is used in traditional medicine in China and India as an antidote for snakebite and insect bites. Also it is used for the treatment of dyspepsia, influenza, dysentery, malaria, and respiratory infections, and as an antipyretic, detoxicant, anti-inflammatory, febrifugal, and antiphlogistic agent. *Andrographis paniculata* has an analgesic property against acute infections of the gastrointestinal tract, respiratory organs, and urinary system. The species is rich in 20-oxygenated flavonoids and labdane diterpenoids (Koteswara et al. 2004). Andro possesses many biological effects such as immune regulation, hepatic protection, cardiovascular protection, antiviral, anticancer, and antidiabetic. In the last decade, several review articles on these activities have been published (Kligler et al. 2006; Akbar 2011; Ghosh et al. 2011; Aromdee 2012; Lim et al. 2012; Zhou et al. 2013; Chua 2014; Wen et al. 2014). It was reported that Andro was able to reduce the lipopolysaccharide (LPS)-induced pro-inflammatory factors such as inducible nitric oxide synthase (iNOS), cyclooxygenase 2 (COX2), prostaglandin E2 (PGE2), and tumour necrosis factor (TNF)-α expression in microglia (Wang et al. 2004). Andro attenuated inflammation by inhibition of necrosis factor (NF-κB) activation through covalent modification of reduced cysteine 62 of p50 (Xia et al. 2004). We have earlier reported that Andro suppressed TNF-induced astrocytic IL-1, IL-6, and TNF (Tzeng et al. 2012). As both microglia and astrocytes have been identified as targets for alleviating allodynia (Watkins and Maier 2003; Ji et al. 2006; McMahon and Malcangio 2009; Gao and Ji 2010a, 2010b), our main interest in the present study was to test if Andro relieves neuropathic pain.

The pain sensation could be roughly divided into three types: nociceptive, inflammatory, and pathological (which includes neuropathic and dysfunctional) pains (Woolf 2010). Neuropathic pain still remains difficult to manage (Burgess and Williams 2010). Central nervous system (CNS) glial cells are involved in pain regulation (Watkins et al. 2001; McMahon and Malcangio 2009). Altering glial activities leads to the release of chemokines, cytokines, protease enzymes, and trophic factors, which in turn modify the excitability of the neuronal elements in the pathway and contribute to central sensitization (McMahon and Malcangio 2009). Activation of mitogen-activated protein kinases (MAPKs) is important signals in astrocytes and in microglia that enforce a positive-feedback loop which feeds on the activation of glial receptors and production of pronociceptive mediators (Ji et al. 2009; Gao and Ji 2010a, 2010b).

Treatment options that could reduce the pain from allodynia include opioids, ion channel blockers, anticonvulsants, antidepressants, topical treatments (lidocaine patch, capsaicin), and ketamine. NSAIDS is used for acute flares of pain (Marcus 2000; Vranken 2009). However, some of the treatments have been outweighed by their side effects, and relief from pain is still not available for about 10% of patients. A combination of treatments may render greater pain relief to certain patients. Therefore, discovery of new drugs that act by a different mechanism is needed.

Allodynia is a pain due to a stimulus (touch or temperature) which does not normally provoke pain. In the spinal cord, the allodynia could be triggered by an autocrine loop mediated by MAPK/nuclear factor kappa-B (NF-κB) pathway which is initiated by pro-inflammatory cytokines (Stellwagen et al. 2005). We used von Frey test to measure the mechanical allodynia behaviour in the spared nerve injury model. Glial fibrillary acidic protein (GFAP) is an astrocyte marker, and upregulation of Glial fibrillary acidic protein immunorectivity (GFAP IR) is an indication of astrocytic responses. Secretion of pro-inflammatory cytokines is a major regulator of central pain sensitization that is of the glial source (Watkins et al. 2001). The expression of pro-inflammatory cytokine, interleukin-1 (IL-1), as well as GFAP as markers for astrocytic activities was tested in the spinal cord to verify if the behaviour was associated with changes in glia activities. These tests examine whether Andro could alleviate allodynia behaviour and establish the correlation of this Andro action to central nervous system (CNS) inflammatory signalling pathways.

## Materials and methods

### Animals

Animals used were male Balb/c mice (weight 30 g, about 8 weeks old). Twenty-six mice were used for the behaviour test, and 17 mice were used for immunohistochemistry. After the operation, animals were kept in individual cages in ventilated, humidity, and temperature-controlled rooms with a 12 h light/dark cycle. They received food pellets and water *ad libitum*. The local Ethical Committee for Animal Research approved all experiments.

### Treatment groups

Experimental groups were as follows: (i) Sham-operated (Sham) group: the skin was incised, and muscle over the sciatic nerves was exposed and sewed back. (ii) Pain control (OP-saline) groups: the mice were operated (for details, see the following) and treated with 150 µL saline intraperitoneally every day. (iii) Andro-treated (OP-Andro) group: the mice were operated and treated with Andro (for details of doses, see the following), which was administered intraperitoneally every day, and behaviour tested on 3, 7, and 14 d. (iv) NSAIDS-treated (OP-NSAIDS) group: the mice were operated and treated with diclofenac salt saline solution (details of doses see following) intraperitoneally every day, and behaviour tested on 3, 7, and 14 d. Six to eight mice were used for each treatment group for three collection dates for behaviour tests (for details, see figure legend). For immunohistochemical study, samples were collected at 7 d. Four to five mice were used for each treatment group at 7 d post-operation (PO).

### Spared nerve injury model for pathological pain model

BalbC mice were subjected to peripheral neuropathy induced by sciatic nerve injury (SNI) (Decosterd and Woolf 2000). The biceps femoris muscle was exposed under isoflurane anaesthesia delivered via a nose cone (2% isoflurane with oxygen as the carrier gas). A section was made through the biceps femoris to expose the sciatic nerve and its three terminal branches: the sural, common peroneal, and tibial nerves. The common peroneal and tibial nerves were sectioned, removing 2–4 mm of the distal nerve stump. Care was taken to avoid touching or stretching the spared sural nerve. Muscle and skin were closed in two separate layers. For sham surgery, the sciatic nerve was exposed as described above but no contact was made with the nerve, on a separate rat other than the one that has branches of sciatic nerve transected. The right side was transected (ipsilateral side, i).

### Behaviour test for allodynia thresholds: von Frey hair test

Mechanical allodynia threshold at the lateral plantar surface of the hind paw was assessed before nerve injury (as basal pain threshold), and then testing began at day 3 after surgery and continued at 7 and 14 d after surgery. Mechanical sensitivity was determined by measuring the withdrawal thresholds, and response percentage to von Frey hairs (Stoelting, Wood Dale, IL) using the up and down method (Dixon 1980) as described by Chaplan et al. (1994), and frequency method by Tanga et al. (2005). Animals were placed in a plastic cage with a wire net floor and were allowed to habituate 10–15 min before commencement of the testing. Animals were also habituated over a period of 2–3 consecutive days by recording a series of baseline measurements. The filaments were applied in an ascending order, five times each at an interval of 2–3 s to the plantar surface of the hind paw (Bourquin et al. 2006) and the smallest filament eliciting a foot withdrawal response was considered as the threshold stimulus. The withdrawal threshold (force of the von Frey hair to which an animal reacts in over 50% of the presentations) was recorded. For measuring response percentage, 0.02 g hair and 0.07 g hair was selected. In three sets of 10 stimulations each when using specific hair, the percentage of withdrawal behaviour exhibited was noted.

### Drug preparation and administration

Lyophilized Andro powder (purchased from Aldrich, Chicago, IL, cat number 365645, 500 mg, 98%) was dissolved in dimethyl sulphoxide (DMSO) and subsequently diluted to a stock of 2.86 mM (1 mg/mL), in 90% normal saline and 10% DMSO. It was administered via an intraperitoneal injection at 0.15 mg in 150 µL saline per animal, based on a calculation from 5 mg/kg animal weight. The NSAIDS diclofenac sodium salt (2-[(2,6-dichlorophenyl)amino]benzeneacetic acid sodium salt) was purchased from Sigma (Chicago, IL) (D6899) and diluted to a stock of 2.86 mM (0.91 mg/mL), in 90% normal saline and 10% DMSO. It was administered via an intraperitoneal injection at 0.15 mg in 165 µL saline per animal, based on a calculation from 5 mg/kg animal weight. The number of molecules administered was less in Andro-treated mice than the diclofenac-treated rats, as the latter had a lower molecular weight. These drugs were administered daily after the operation. Thereafter, the mice were allowed 1.5 h to recuperate before the first injection.

### Collection of tissue samples

To localize spinal glia, mice were deeply anaesthetized with sevoflurane and perfused transcardially with saline followed by 4% paraformaldehyde in 0.1 M phosphate buffer. The L4–L6 segment of the spinal cord was dissected out, post-fixed in 4% paraformaldehyde solution for 2 h at 4 °C, and transferred to a series of a solution of phosphate-buffered saline **(**PBS), graded ethanol/PBS, xylene, graded xylene/wax mixture, and finally embedded in wax. Tissues were transverse sectioned at 3–4 µ and mounted onto silane-coated glass slides.

### Immunohistochemistry

Wax sections were de-waxed in a series of xylene, graded xylene/ethanol mixture, graded ethanol mixture, and finally incubation in PBS. The antigen was recovered using Antigen retrieval solution (R&D Systems, Inc., Minneapolis, MN). Sections were then left to incubate with primary antibodies overnight at 4 °C. Primary antibodies used were goat anti-interleukin 1 β (1:250, IL-1β, AF-501-NA, R&D Systems, Inc., Minneapolis, MN), GFAP (1:1000, Dako, Carpinteria, CA). Secondary antibody for fluorescent photography was rabbit anti-goat IgG conjugated to Alexa 488 (Molecular Probes, Eugene, OR) used at 1:800. For some antibodies, ABC kit (Vector Laboratory, Burlingame, CA) was used and chromogen developed, according to the suggestion from the manufacturer. Primary antibody omission controls were used for all immunostaining protocols to control non-specific binding. Fluorescent or chromagen photography was performed with a Zeiss Axioscope microscope (Carl Zeiss Meditec, Inc., Dublin, CA) equipped with charge coupled device (CCD) camera with appropriate filter sets. Haematoxylin and eosin (H&E) solution were purchased from Muto Pure Chemical Co. Ltd (Tokyo, Japan).

### Quantification of immunoreactivity (IR)

IR was analyzed by using chromagen or fluorescence intensity of the photography from 4 to 5 independent animals of each treatment group. Photographic images were acquired using the same setting for each experiment for the quantitative comparison. All pixels within the area to be measured were analyzed with ImageJ software (NIH Image J system, Bethesda, MD) on a 0 (black) to 255 (white) scale. The number of pixels that had a value of less than 50 (for chromagen sample) or larger than 120 (for fluorescence intensity) was calculated and used to estimate the IR in the region of interest.

### Statistical methods

In this study, there were 12 time–condition sets of experiments formed by different combinations of three time-points (days 3, 7, and 14) and four conditions (left, right, 0.02 g, and 0.07 g). For a convenience, ‘withdrawn threshold’ and ‘response percentage’ were taken as ‘response score.’ The response scores among different treatments e.g., Andro, NSAIDs, and saline were statistically compared within each time-point set under different conditions (see the following).

Within a time–condition set of an experiment, one-way ANOVA model was fitted to the response score data, and the normality assumption of residuals of the model was tested using the Kolmogorov–Smirnov test and the Shapiro–Wilk test. The Kruskal–Wallis test (a non-parametric one-way ANOVA testing method) was conducted to determine whether there were any significant differences at the response score levels among the different treatment groups. Next, multiple comparisons, including the response score levels between Andro and saline, Andro and NSAIDs, and NSAIDs and saline, were made. This was done within each time–condition set using the one-tailed Wilcoxon rank-sum test to check the comparative effectiveness between Andro and saline, Andro and NSAIDs, and NSAIDs and saline. The *p* values of multiple comparisons were adjusted within each time–condition set and in the IL1 study using the method proposed by Hochberg (1988). All the hypothesis tests in this study were based on the significant level of 0.05 and were proceeded with R 3.32 (R Core Team 2017).

Among 12 time–condition categories, eight *p* values of Kolmogorov–Smirnov tests and seven *p* values of Shapiro–Wilk tests were less than 0.05, which indicated the normality assumption of residuals was not adequate for more than half of the time–condition categories, and the conventional parametric one-way ANOVA and the two-sample *t*-test were not applicable to hypothesis tests in these categories. For consistency, the Kruskal–Wallis test and the Wilcoxon rank-sum test were used for all one-way ANOVAs and all two-sample comparisons, respectively (Please refer Supplementary material).

## Results

### The withdrawal thresholds of the ipsilateral hind paw in the OP-Andro group were found to be significantly increased compared with OP-saline group and OP-NSAIDs group

**Figure 1.**
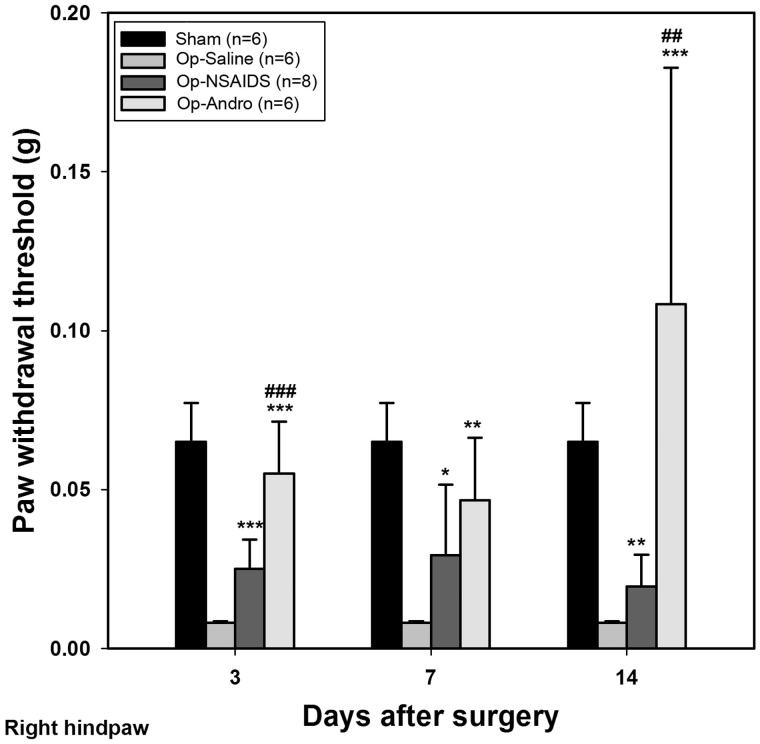
Withdrawal threshold for ipsilateral paw. Mechanical allodynia threshold was assessed by von Frey test for mice with different treatments. The smallest filament eliciting a foot withdrawal response was considered the threshold stimulus. The withdrawal threshold was set at the point where out of 10 test over 50% of the time the paw moved. Sham: mice in the Sham group, with only the skin cut and sutured. OP-saline: mice had their right sciatic nerves transected, and injected daily with saline. OP-NSAIDs: mice had their right sciatic nerves transected, and injected daily with diclofenac solution. OP-Andro: mice had their right sciatic nerves transected, and injected daily with andrographolide solution. The right hind paw withdrawal thresholds were averaged within groups. OP-Andro group withdrawal thresholds were significantly increased compared with the OP-saline group (*) and the OP-NSAIDs group (#). * or # : *p* < 0.05, ** or ## : *p* < 0.01, *** or ### : *p* < 0.005.

The withdrawal threshold was set at the point where out of 10 tests, over 50% of the time, the paw moved. Three days after the operation, the OP-saline group showed a significant decrease in allodynia threshold. With repeated treatment of diclofenac solution, the withdrawal threshold increased significantly compared with the OP-saline groups (OP-NSAIDS versus OP-saline, 3–14 d in Figure 1). The OP-Andro group showed increased threshold compared with the OP-saline group (OP-Andro versus OP-saline at 3, 7, and 14 d, Figure 1), and also compared with the OP-NSAIDS group (OP-Andro versus OP-NSAIDS at 3, 7, and 14 d, Figure 1). The ratio of threshold between the OP-Andro and the OP-saline group was 13.54, and that for OP-Andro/OP-NSAIDS was 5.56, in the right hind paw at 14 d.

### The withdraw thresholds of contralateral hind paw in the OP-Andro group were significantly increased compared with OP-saline group and OP-NSAIDs group, respectively

**Figure 2.**
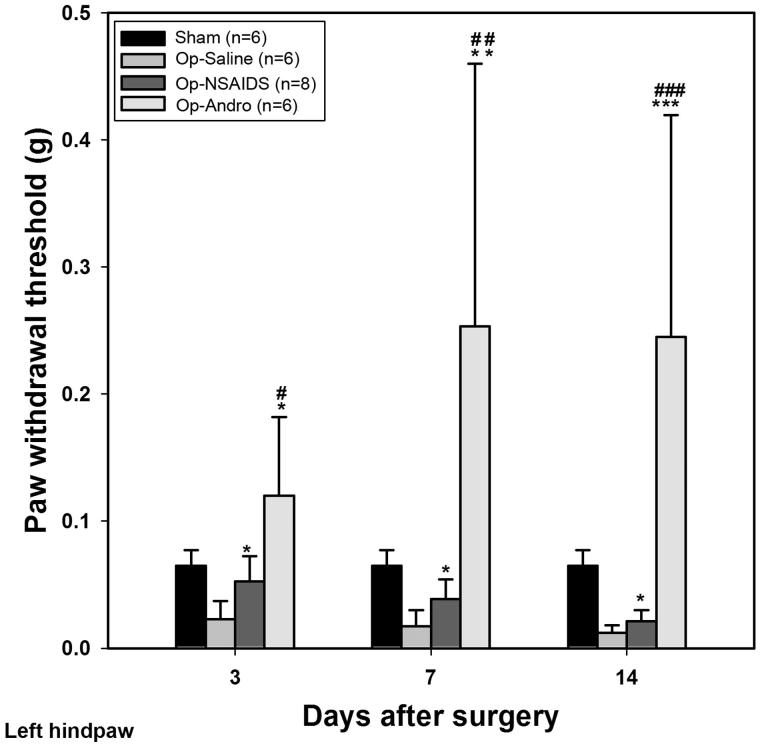
Withdrawal threshold for contralateral paw. Mechanical allodynia threshold was assessed by von Frey test for mice with different treatments. The smallest filament eliciting a foot withdrawal response was considered the threshold stimulus. The withdrawal threshold was set at the point where out of 10 test over 50% of the time the paw moved. Sham: mice in the Sham group, with only the skin cut and sutured. OP-Saline: mice had their right sciatic nerves transected, and injected daily with saline. OP-NSAIDs: mice had their right sciatic nerves transected, and injected daily with diclofenac solution. OP-Andro: mice had their right sciatic nerves transected, and injected daily with andrographolide solution. The left hind paw withdrawal thresholds were averaged within groups. OP-Andro group withdrawal thresholds were significantly increased compared with the OP-saline group (*) and the OP-NSAIDs group (#). * or # : *p* < 0.05, ** or ## : *p* < 0.01, *** or ### : *p* < 0.005.

These data showed the contralateral side of withdrawal responses. We observed reduced threshold compared with the Sham-group in the left paw of the OP-saline group, although not to the same extent as the right side (withdrawal thresholds in the right and left paws were = 0.01 and 0.02, respectively) (Figure 2). Diclofenac solution increased the withdrawal threshold to a level significantly different to the OP-saline group at 3, 7, and 14 d PO (OP-NSAIDs versus OP-saline, Figure 2**)**. However, Andro treatment further increased the threshold significantly compared with the diclofenac treatment (OP-Andro versus OP-NSAIDS, Figure 2). This trend continued through 7 and 14 d after the operation. The ratio of threshold between the OP-Andro and the OP-saline group was 20.42, and that for OP-Andro/OP-NSAIDS was 11.67, in the left hind paw, at 14 d. The threshold levels in the contralateral side varied significantly, and the standard deviation of the curve was large from the 3rd to the 14th day.

### Op-Andro group showed lower withdrawal rate than theOP-saline group and the OP-NSAID group

**Figure 3.**
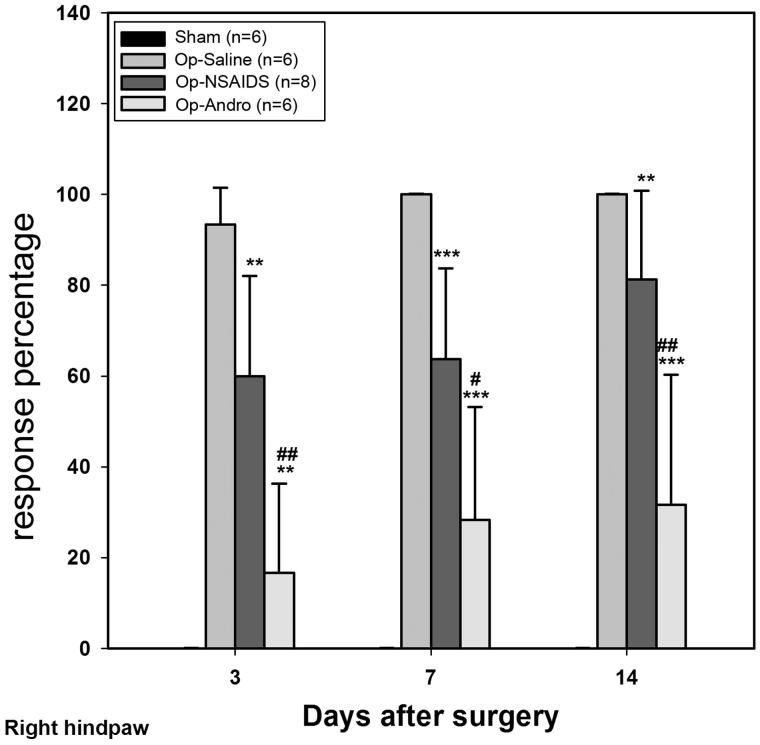
The response percentage of the von Frey test (ipsilateral). Hair (0.02 g) was used for this measurement. For each 10 tests when using specific hair, the percentage of withdrawal behaviour exhibited was noted. The right hindpaw response percentages were averaged within groups. Sham: mice in the Sham group, with only the skin cut and sutured. OP-saline: mice had their right sciatic nerves transected, and injected daily with saline. OP-NSAIDs: mice had their right sciatic nerves transected, and injected daily with diclofenac solution. OP-Andro: mice had their right sciatic nerves transected, and injected daily with andrographolide solution. OP-Andro group response percentage was significantly decreased compared with the OP-saline group (*) and the OP-NSAIDs group (#). * or # : *p* < 0.05, ** or ## : *p* < 0.01, *** or ### : *p* < 0.005.

**Figure 4.**
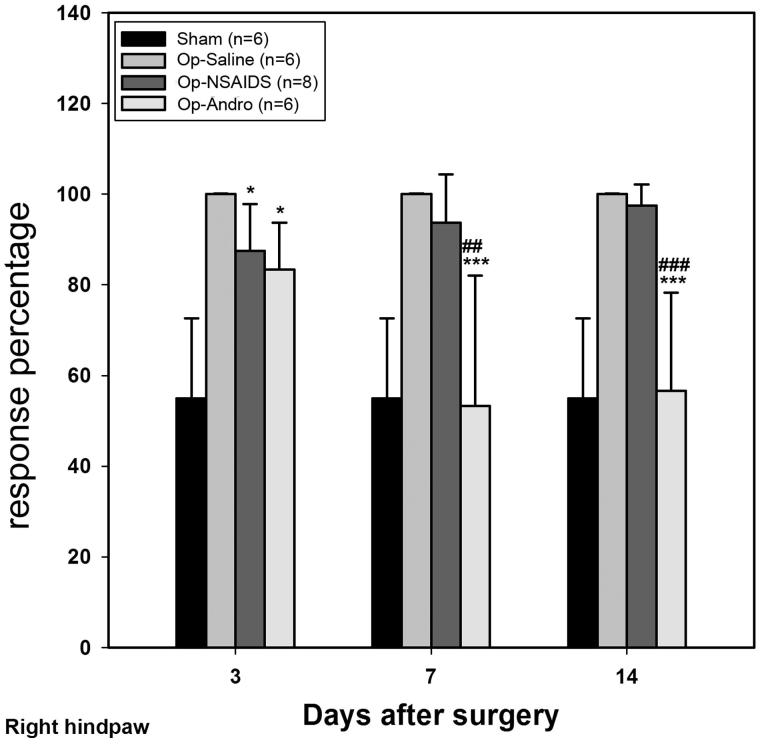
The response percentage of the von Frey test (ipsilateral). Hair (0.07 g) was used for this measurement. For each 10 tests when using specific hair, the percentage of withdrawal behaviour exhibited was noted. The left hindpaw response percentage was averaged within groups. Sham: mice in the Sham group, with only the skin cut and sutured. OP-Saline: mice had their right sciatic nerves transected, and injected daily with saline. OP-NSAIDs: mice had their right sciatic nerves transected, and injected daily with diclofenac solution. OP-Andro: mice had their right sciatic nerves transected, and injected daily with andrographolide solution. OP-Andro group response percentages were significantly decreased compared with the OP-saline group (*) and the OP-NSAIDs group (#). * : *p* < 0.05,*** or ### : *p* < 0.005.

von Frey hairs (0.02 g and 0.07 g) were selected, and the respective withdrawal rates were analyzed. The withdrawal rate was determined by the following formula: (number of withdrawal)/10) × 100%. Lower withdrawal rate indicates less mechanical allodynia. When the 0.02 g hair was used (Figure 3), no response evoked in the paws of the sham-group mice. After the operation, if treated only with saline, the paw moved nearly every time during the three time points tested, which indicates a strong allodynia behaviour (OP-saline group). When treated with diclofenac (OP-NSAIDs group), the withdrawal rate was significantly reduced. When treated with Andro (OP-Andro), the withdrawal rate was further reduced (cf. OP-NSAIDS) (Figure 3). The ratio of response percentage for OP-Andro/OP-saline and for OP-Andro/OP-NSAIDS was 32% and 39% at 4 d, respectively. For the 0.07 g hair, after the operation, if treated only with saline, the paw moved nearly every time during the three time points tested similar to that of 0.02 g hair. When treated with diclofenac (OP-NSAIDs group), the withdrawal rate was significantly reduced at the acute stage (3 d), but not afterward (7 and 14 d). If treated with Andro (OP-Andro), the withdrawal rate was significantly reduced compared with both the saline group and the NSAIDS group (Figure 4) (7 and 14 d). The ratio of response percentage for OP-Andro/OP-saline and for OP-Andro/OP-NSAIDS was.57% and 58% at 0.07 g, respectively. These data also demonstrated that the NSAIDS group showed lower withdrawal rate than the OP-saline group only at 0.02 g but not at longer time point for 0.07 g von Frey hair weight scale (the ratio of response percentage for NSAIDS/OP-saline at 7 d was 64% for 0.02 g and 98% for 0.07 g).

### GFAP IR at day7 PO

**Figure 5.**
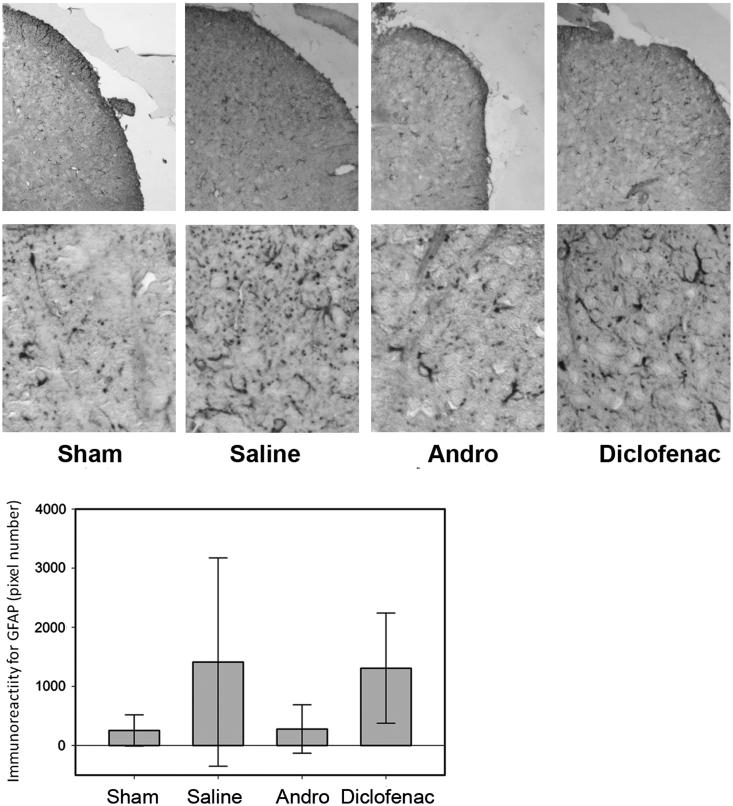
GFAP immunoreactivity in dorsal horn. The mice were sacrificed by intracardiac perfusion. L4–L6 spinal segments were collected 7 d after operation, and wax sectioned at 4 μm. The astrocytes were immmunostained with GFAP and detected by colour chromogen by vector ABC kit. The sections were photographed and digital images capture astrocytes in the dorsal horn analyzed by image J (NIH Image J system, Bethesda, MD). The upper panel showed the part of the spinal cord where the photos were taken. The lower panel showed the morphology of astrocytes stained by GFAP. The variations among mice were too large for the results to be statistically significant. Details of treatment groups are similar to that of Figures 1–4.

Following an earlier report by Tsuda et al. (2011), day 7 PO was selected as the reference point for measuring the difference in GFAP IR and astrocytic morphology in the dorsal horn of L4–L6 spinal segments, for post-peripheral injury proliferation of astrocytes peaks at 4–7 d PO. Due to the large variation among the staining results, the difference among the groups was not statistically significant. Representative GFAP IR is shown in Figure 5. In some mice of the OP-saline groups, the astrocytes showed more thickened branches, and many more cross-sections of the GFAP-positive processes in the shape of small dots in the dorsal horn area. While, some mice in the Andro-treated group, the processes of GFAP + cells were thin with fewer branches, and limited cross-sections of the GFAP + branches. The GFAP IR was quantitatively analyzed, and the IRs for OP-Andro and OP-NSAID groups were not significantly reduced compared with the OP-saline group (Figure 5).

### IR for proinflammatory cytokines IL-1 was lower in theOP-Andro group compared with the OP-saline groupat day-7 PO

**Figure 6.**
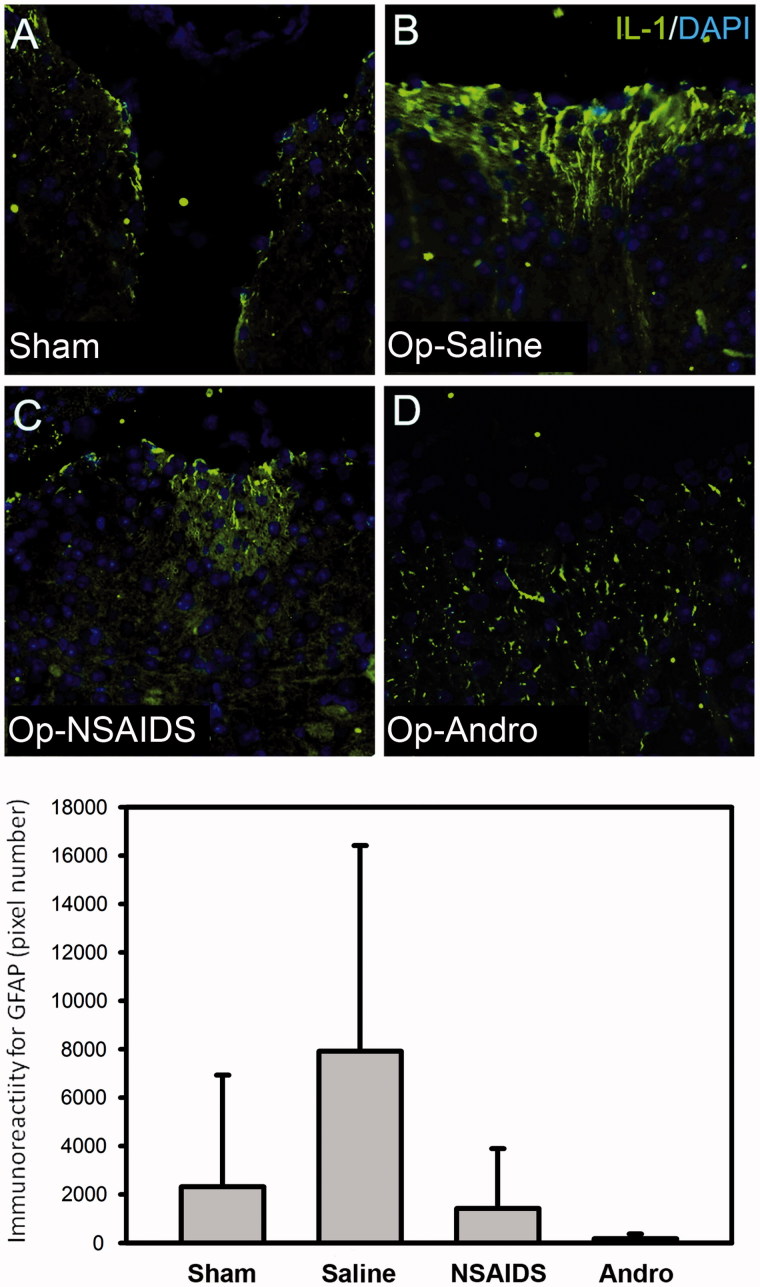
IL-1 immunoreactivity in dorsal horn. The mice were sacrificed by intracardiac perfusion. L4–L6 spinal segments were collected 7 d after operation, and wax sectioned at 4 μm. The IL-1 level (green fluorescence) was up-regulated in the L4–L6 spinal segment in the OP-saline group (B), as expected. The IL-1 level in the OP-NSAIDS group (C) and the OP-Andro group (D) were significantly lower than the OP-saline group (B). Details of treatment groups are similar to that of Figures 1–4.

The IL-1 level was upregulated in the L4–L6 spinal segments in the OP-saline group as expected (Figure 6). The elevated IL-1 IR was found in cellular process within the dorsal aspect of the spinal cord. The IL-1 level in the OP-Andro group (D) was lower than the OP-saline group (B) (Figure 6). The reduction in OP-NSAIDS was not significant, but there was a trend of reduction (*p* = 0.14 adjusted *p* values of Wilcoxon rank-sum tests for multiple comparisons).

In summary, the left and right hind paw withdrawal thresholds of the OP-Andro group were found to be significantly increased compared with OP-saline and OP-NSAIDs groups. Withdrawal thresholds of the OP-NSAIDS group were increased compared with the OP-saline group at 3 and 14 d. The withdrawal rate was lower in the OP-Andro group than in the OP-saline and OP-NSAIDs groups at two different weight scales (0.02 g and 0.07 g), especially at a longer period. The withdrawal rate was lower in the OP-NSAIDS group compared with the OP-saline group of 0.02 g weight scale at all three time points, but only at 3 d for 0.07 g weight scale. The data indicate that Andro alleviated mechanical allodynia more than NSAIDS when the same concentration was used. The reduced allodynia for Andro was accompanied by lower proinflammatory cytokines IL-1 levels in the dorsal horn area.

## Discussion

### Andro alleviate pain and mirror pain, and better than NSAIDS

Our data showed that the thresholds for inducing allodynia were increased, while response percentage decreased, for the Andro group, compared with the saline group in the right hind paw. This indicated that Andro reduced the neurological pain behaviour. The Andro group also showed increased threshold and decreased response percentage compared with the NSAIDS group. In our study, an equal amount in weight percentage (5 mg/kg) was used for both NSAIDS and Andro. As the molecular weight of Andro is higher than the diclofenac salt, more diclofenac salt was used than the Andro group, an indication that Andro could be even better than diclofenac salt if the equal molar concentration is injected.

As the nerve injury was executed at the right sciatic nerve, one might expect no response in the contralateral side. However, peripheral nerve injury has been known to produce central sensitization, which means pain sensation evoked by touch alone could occur in the non-operated site. Our data from the left paw suggested that Andro was more effective for alleviating centrally sensitized pain (mirror pain) than NSAIDS, especially in the chronic state.

### What's new from our spared nerve model data?

Analgesic effect was reported for Andro previously by the acetic acid-induced writhing and the hot-plate tests (Suebsasana et al. 2009; Sulaiman et al. 2010). The effective doses that were tested previously were 10–50 and 4–8 mg/kg, and were similar to our effective dose (5 mg/kg). These two animal models developed pain within seconds or minutes after administration of the chemicals or temperature stimulus, and test for the alleviation of acute pain sensation, which may not have been as much aggravated within the CNS components. The injured sciatic nerve pain model, which we used, more closely mimic the pathological and chronic pain that patients encounter with trauma of the limb, bone, or the central nervous system. Therefore, our data further contribute to the understanding of the analgesic effect of andrographolide by demonstrating its effect for pathological or chronic pain, which still remains a serious concern in pain management.

### Astrocytic activities may not be the key regulators of the Andro effect

Due to a limited sample size, and that the data obtained *in vivo* vary greatly, the statistics were not significantly different between Andro and saline-treated groups (adjusted *p* values = 0.11, Wilcoxon rank-sum tests for multiple comparisons). Although ours and other reports have demonstrated that andrographolide reduced proinflammatory cytokines IL-1, IL-6, and TNF-α and GFAP IR in stimulated primary astrocytes (Tzeng et al. 2012; Wong et al. 2014), the present data do not corroborate that astrocytic activities are sufficient to regulate Andro's effect on allodynia behaviour.

It is reasonable that results from *in vitro* experiments do not always match with observations *in vivo*. Although astrocytes serve as an important mediator for pain relay and modulation (Hofstetter et al. 2005; Gao and Ji 2010b); however, the actual pain sensation itself is complex, and several factors contribute to it. It is possible that summation of astrocytic activities and other factor(s) co-contribute to the final behavioural outcome. Further, we observed an increased withdrawal threshold for mirror pain in Andro-treated mice than the Sham group at 7 and 14 d in some mice. It means that not only the chronic pain, which is exacerbated by glial inflammation response, but also the basal pain sensation, or even sensation per se, may be influenced by Andro; however, further research is needed to verify this hypothesis.

### Andro out-performed diclofenac

We compared the present diclofenac salt results with the report of Dong et al. (2013), where ketorac (sub-type of NSAIDS) was used. They found that ketorac reduced the activities of dorsal horn astrocyte (Dong et al. 2013). However, while doing this comparison, we realized that the diclofenac salt concentration used in our study was less. Also, our injection method may have result in further diffusion, while in other’s case, it was more localized and near CNS. As the effect in our study was just on the borderline of reducing pain, hence the discrepancy in effective reduction at all three time-points at 0.02 g, but only at the acute time point (3 d) and not at 7 and 14 d (Figures 3 and 4). This corroborates the results that diclofenac in our study did not significantly reduce the IL-1 level or astrocytic response at 7 d PO. Andro, on the contrary, was applied at the same concentration and the same method as of diclofenac, but was good at the longer time period (7 and 14 d). This demonstrates the advantages of Andro for reducing pathological pain.

### Proposed mechanism

**Figure 7.**
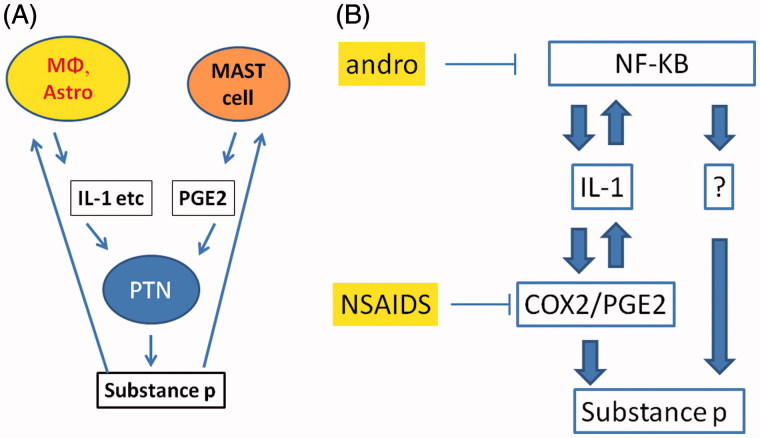
(A) Diagram demonstrating the simplified view of glia secreted IL-1 and PGE2 promotes substance P and pain sensation, which provide a positive feedback to the glia, fuelling more IL-1 and PGE2. (B) Proposed mechanism of action of Andro and NSAIDS. For details, see Discussion section.

Central glial (astrocyte and microglia as functional entities) regulates the central sensitization of pain transmission. The glial cells form a positive-feedback loop with the post-traumatic neuron (PTN), by releasing pro-inflammatory factors that excite PTN to release pain substance followed by reverse action on glia to elicit more pro-inflammatory factors (Watkins et al. 2001) (Figure 7(A)). Blocking of IL-1 activity decreases inflammation-induced central PGE2 levels and mechanical hyperalgesia (Samad et al. 2001; Gabay et al. 2011). On the one hand, one of the IL-1 upstream regulators is NF-κB, which forms a positive-feedback loop with the IL-1 (Smith et al. 2006). On the other hand, IL-1 regulates the *COX* genes, and hence the PEG2 level and the execution of the inflammatory response. NF-κB, IL-1, and COX2-regulated PEG2 was in molecular regulatory circuits that positively feed into substance P and pain sensation (Figure 7(B)). We have demonstrated that there was a correlation of IL-1 down-regulation, and the behavioural change in the Andro-treated group. Andro acts as a NF-κB inhibitor in the wide range of cells (Xia et al. 2004; Hidalgo et al. 2005; Wang et al. 2007; Bao et al. 2009). The down-regulation of IL-1 observed for the Andro-treated group supports an assumption that Andro's anti-allodynia effect acts via its NF-κB antagonism. However, the fact that Andro has a higher effect than NSAIDS especially at the longer time point (7 and 14 d) indicated that Andro might have acted on molecules other than pro-inflammatory cytokines that were within the NF-κB-IL-1 PEG2 pathways (Figure 7).

## Summary

In summary, Andro reduced allodynia in mice with spared injured sciatic nerves. The effect was noted for the operated and for the mirror pain site. This reduced allodynia behaviour outperformed diclofenac treatment, especially at time-points of 7 and 14 d. The reduced IL-1 level was observed in the Andro-treated groups. Andro may be further developed as a drug, alone or in combination with other drugs for the relief from pathological pain.

## Supplementary Material

Yi-Lo_Lin_et_al_supplemental_content.zip
