# Supplementary material for: Andrographolide relieved pathological pain generated by spared nerve injury model in mice
Source: Pharm Biol. 2018 Feb 1;56(1):124–31. doi: 10.1080/13880209.2018.1426614 (PMC6130553; doi:10.1080/13880209.2018.1426614)
Supplement: Yi-Lo_Lin_et_al_supplemental_content.zip [file IPHB_A_1426614_SM6391.zip › Yi-Lo Lin et al supplemental content.pdf]

Supplementary File (Statistical Analysis)

Statistical Method

In this study, there were twelve time-condition set of experiments formed by different combinations of three time-points (day 3, day 7, and day 14) and four conditions (left, right, 0.02g, 0.07g). For convenience, 'withdrawal threshold' and 'percentage response' were taken as 'response score.' The response scores among different treatments e.g. Andro, NSAIDs, and Saline were statistically compared within each time-point set under different conditions.

Within a time-condition set of experiment, one-way ANOVA model was fitted to the response score data, and the normality assumption of residuals of the model was tested using the Kolmogorov-Smirnov and the Shapiro-Wilk tests. The Kruskal-Wallis test (a non-parametric one-way ANOVA testing method) was conducted to determine whether there were any significant differences among the response score levels among different treatment groups. Next, multiple comparisons, including the response score levels between Andro and Saline; Andro and NSAIDs, and NSAIDs and Saline were made. This was done within each time-condition set using the one-tailed Wilcoxon rank-sum test to check the comparative effectiveness between Andro and Saline, Andro and NSAIDs, and NSAIDs and Saline.

For the study of IL1 data, the one-tailed Wilcoxon rank-sum test was also used to decide whether IL1 level of Andro group was smaller than that of Saline, NSAIDs and Saline groups.

Treatment comparison for GFAP also used one-tailed Wilcoxon rank-sum test to decide whether GFPA level of Andro, NSAIDs, and Sham were better than that of Saline.

The p-values of multiple comparisons were adjusted using the method proposed by Hochberg (1988). All the hypothesis tests in this study were based on the significant level of 0.05 and were based on R 3.32 (www.r-project.org).

Statistical Results

Table1:

	Test	Day 3	Day 7	Day 14
Left	Kolmogorov-Smirnov	0.0545	0.0001	0.0001
	Shapiro-Wilk	0.0098	0.0003	0.0008
	Kruskal-Wallis	0.0060	0.0012	0.0007
Right	Kolmogorov-Smirnov	0.0020	0.0351	0.0001
	Shapiro-Wilk	0.0062	0.2548	0.0063
	Kruskal-Wallis	0.0002	0.0052	0.0008
0.02g	Kolmogorov-Smirnov	0.4698	0.2805	0.0100
	Shapiro-Wilk	0.1011	0.0901	0.0878
	Kruskal-Wallis	0.0008	0.0006	0.0007
0.07g	Kolmogorov-Smirnov	0.0869	0.0012	0.0000
	Shapiro-Wilk	0.3570	0.0160	0.0006
	Kruskal-Wallis	0.0123	0.0010	0.0004

p-values of normality tests (Kolmogorov-Smirnov test and Shapiro-Wilk test) of residuals and Kruskal-Wallis one-way ANOVA test.

Among 12 time-condition sets of experiments, 8 p-values of Kolmogorov-Smirnov tests and 7 p-values of Shapiro-Wilk tests were less than 0.05, i.e., the normality assumption of residuals was not adequate for more than half of the time-condition sets; therefore, the conventional parametric one-way ANOVA test and the two-samples t-test were not applicable to hypothesis tests in these sets. For consistency, the Kruskal-Wallis test and the Wilcoxon rank-sum test were used for all multi-sample comparisons and all two-sample comparisons, respectively.

All p-values of the Kruskal-Wallis one-way ANOVA of different time-condition sets located between 0.0004 and 0.006 except for day 3 (0.07g) groups, of which p-value was 0.0123. These results provide strong evidence that the effects of three treatments were very different.

The adjusted p-values of the Wilcoxon rank-sum tests for multiple comparisons within each time-condition sets have been shown in Table 2. The results indicated that Andro's effects were better than Saline's effects under all conditions. While, Andro's effects were better than NSAIDs' effects under two-thirds of all conditions, and NSAID's effects were better than the Saline's effects also under two-thirds of all conditions. Even in case of time-condition sets the Wilcoxon rank-sum tests did not yield significant evidence of Andro's effects being better than NSAIDs' effects, or NSAIDs' effects being better than Saline's effects. Their p-values were still at

borderline except for those of 0.07g sets. The mean levels of the treatment response score in **Table 3** consistently showed the trend that Andro was more effective than NSAIDs, and NSAIDs was more effective than Saline.

IL1 study indicated that IL1 level of Andro group was significantly smaller than that of Saline group. Although the Wilcoxon rank-sum results of testing IL1 of Andro being smaller than IL1 of NSAIDs, and testing of IL1 of NSAIDs being smaller than IL1 of saline did not provide statistical significance, the means IL1 levels of these three treatments did show the trend (**Table 4, 5**).

For GFAP, there were trends of Andro and Sham being better than Saline (marginally significant with non-adjusted p-value 0.057 and 0.056, respectively, although the adjusted p-values for both were slightly over 0.1) (**Table 6, 7**).

Table2:

	Wilcoxon Rank-Sum Test	P-value Type	Day 3	Day 7	Day 14
Left	Andro vs. NSAIDs	p-value	0.0455	0.0034	0.0009
		adjusted p-value	0.0769	**0.0343**	**0.0160**
	Andro vs. Saline	p-value	0.0049	0.0028	0.0020
		adjusted p-value	**0.0354**	**0.0307**	**0.0260**
	NSAIDs vs. Saline	p-value	0.0093	0.0101	0.0236
		adjusted p-value	**0.0504**	**0.0504**	0.0708
Right	Andro vs. NSAIDs	p-value	0.0023	0.0769	0.0043
		adjusted p-value	**0.0273**	0.0769	**0.0354**
	Andro vs. Saline	p-value	0.0012	0.0013	0.0014
		adjusted p-value	**0.0189**	**0.0189**	**0.0191**
	NSAIDs vs. Saline	p-value	0.0005	0.0141	0.0051
		adjusted p-value	**0.0086**	0.0564	**0.0354**
0.02g	Andro vs. NSAIDs	p-value	0.0033	0.0179	0.0039
		adjusted p-value	**0.0327**	0.0718	**0.0350**
	Andro vs. Saline	p-value	0.0022	0.0013	0.0013
		adjusted p-value	**0.0260**	**0.0177**	**0.0177**
	NSAIDs vs. Saline	p-value	0.0075	0.0008	0.0061
		adjusted p-value	**0.0376**	**0.0130**	**0.0363**
0.07g	Andro vs. NSAIDs	p-value	0.2299	0.0027	0.0007
		adjusted p-value	0.2299	**0.0297**	**0.0124**
	Andro vs. Saline	p-value	0.0045	0.0014	0.0011
		adjusted p-value	**0.0360**	**0.0177**	**0.0177**
	NSAIDs vs. Saline	p-value	0.0059	0.0630	0.1213
		adjusted p-value	**0.0363**	0.1890	0.2299

Table3:

		Day3		Day7		Day14	
		mean	sd	mean	sd	mean	sd
Left	Andro	0.1200	0.0620	0.2533	0.2066	0.2450	0.1743
	NSAIDs	0.0525	0.0198	0.0388	0.0155	0.0210	0.0088
	Saline	0.0227	0.0145	0.0173	0.0126	0.0120	0.0062
Right	Andro	0.0550	0.0164	0.0467	0.0197	0.1083	0.0744
	NSAIDs	0.0250	0.0093	0.0293	0.0222	0.0195	0.0099
	Saline	0.0080	0.0000	0.0080	0.0000	0.0080	0.0000
0.02g	Andro	16.6667	19.6638	28.3333	24.8328	31.6667	28.5774
	NSAIDs	60.0000	22.0389	63.7500	19.9553	81.2500	19.5941
	Saline	93.3333	8.1650	100.0000	0.0000	100.0000	0.0000
0.07g	Andro	83.3333	10.3280	53.3333	28.7518	56.6667	21.6025
	NSAIDs	87.5000	10.3510	93.7500	10.6066	97.5000	4.6291
	Saline	100.0000	0.0000	100.0000	0.0000	100.0000	0.0000

Table4:

IL1	p	adjusted-p
Andro < Saline	0.0079	0.0238
Andro < NSAIDs	0.8000	0.8000
NSAIDs < Saline	0.0714	0.1429

Table5:

IL1	mean	sd
Saline	7917.80	8501.45
NSAID	1426.67	2469.33
Andro	177.75	191.26

Table 6:

GFAP	p	adjusted p
Sham > Saline	0.0556	0.1143
Andro < Saline	0.0571	0.1143
NSAIDs <Saline	0.3429	0.3429

Table 7:

GFAP	mean	sd
Sham	255.20	264.59
Saline	1764.25	1817.49
Andro	279.50	409.85
NSAIDS	1307.75	931.07
